# Vertebral Body Avascular Osteonecrosis: An Unusual Late Complication of Tracheostomy

**DOI:** 10.7759/cureus.22603

**Published:** 2022-02-25

**Authors:** Alexander Rozov, Valentina Dubov, Evgenia Cherniavsky, Eli Magen

**Affiliations:** 1 Internal Medicine E, Barzilai University Medical Center, Ashkelon, ISR; 2 Radiology, Barzilai University Medical Center, Ashkelon, ISR; 3 Internal Medicine C, Barzilai University Medical Center, Ashkelon, ISR

**Keywords:** osteonecrosis, avascular, tracheostomy, tube, endotracheal, leak, cuff

## Abstract

Vertebral body erosion is a rare late complication of tracheostomy. Here we present the case of a 30-year-old female patient, in a vegetative state after severe brain injury, with a permanent tracheostomy and prolonged mechanical ventilation, who suffered from recurrent episodes of severe air leakage with oxygenation drop due to a puncture in the tracheostomy tube cuff. A neck computed tomography (CT) detected destruction of two vertebral bodies, C7 and Th1, and a bony fragment - a remnant of C7 penetrated the trachea and probably repetitively punctured the inflated cuff. A biopsy of the C7 vertebral body was performed under CT guidance to rule out osteomyelitis. The biopsy revealed necrotic bone spicules surrounded by vascular-rich fibrous tissue, without evidence of inflammation. C7 vertebral body avascular osteonecrosis was diagnosed. The case highlights the importance of monitoring cuff pressure during long-term use of cuffed endotracheal tubes to avoid hyperinflation and subsequent ischemic complications.

## Introduction

Tracheostomy can be associated with several late complications, such as acute obstruction, granuloma, trachea-brachiocephalic artery fistula, and death [1]. Vertebral body erosion is another rare late complication of tracheostomy [2]. Here we describe an unusual case of vertebral body osteonecrosis, which was diagnosed after a repetitive deflation of the tracheostomy tube cuff by the penetrating residual C7 vertebral fragments. 

The authors obtained the signed authorization of the subject’s legally authorized representative to use the subject’s information in the article. As a case report does not meet the definition of “research,” the Barzilai University Medical Center Institutional Review Board on human research gave an exemption for the study approval.

## Case presentation

A 30-year-old female patient, in a vegetative state after severe brain injury, with a permanent tracheostomy and prolonged mechanical ventilation was admitted to our hospital due to recurrent aspiration pneumonia.

At the age of 25, the patient, previously healthy, was involved in a motor vehicle accident and sustained a heavy brain injury, which left her in a permanent vegetative state with a percutaneous endoscopic gastrostomy feeding tube. For five years, the patient was mechanically ventilated with a permanent tracheostomy.

A few months before, after recurrent episodes of aspiration pneumonia, her air-inflated cannula was changed to a water-inflated balloon cannula to prevent future aspirations. The patient was also diagnosed with tracheomalacia. 

Due to severe hypersalivation botulinum toxin was injected into her salivary glands. Despite all efforts, the patient continued to suffer from episodes of abrupt saturation drops.

The patient’s caregiver was instructed by the attending pulmonologist to add water to the balloon whenever a leakage of air from the mouth was evident. It is plausible that the practice of adding water to the balloon caused increased and uncontrolled pressure on the trachea and surrounding tissues.

During the patient’s last hospitalization due to an additional episode of aspiration pneumonia, the medical staff noted three episodes of severe air leakage with oxygenation drop due to a puncture in the tracheostomy tube cuff. All these episodes occurred within one week.

**Figure 1:**
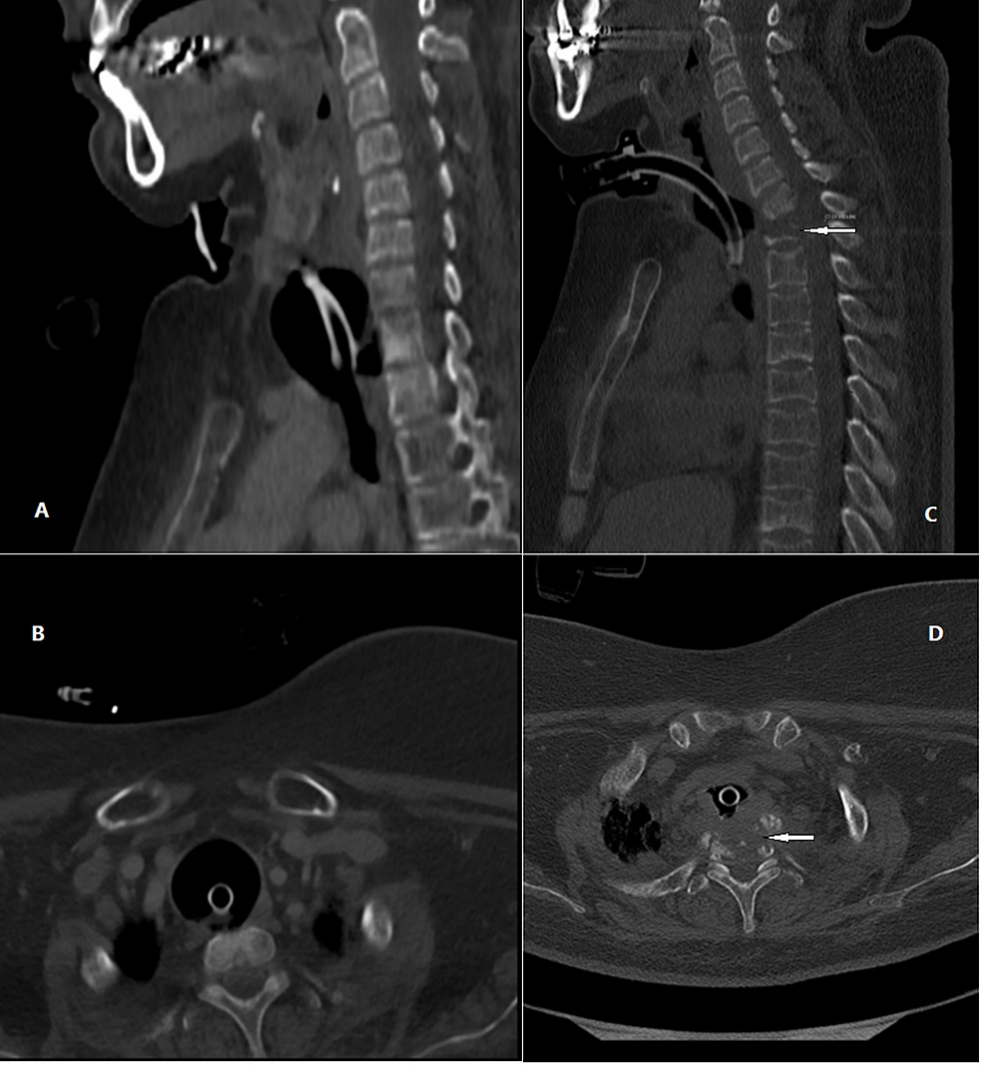
Neck CT images (A) Sagittal view neck CT image. (B) Axial view neck CT image. The neck CT scan was performed five months before vertebral destruction. CT scan demonstrates the normal shape of vertebral bodies with mild reactive sclerosis and minimal erosion in the anterior aspect of Th1. (C) Sagittal view neck CT image. (D) Axial view neck CT image. The neck CT scan was performed during hospitalization. CT scan demonstrates the destruction of two vertebral bodies, C7 and Th1 (arrows). CT, computed tomography.

Since a cuff rupture is a rare occurrence, a neck computed tomography (CT) was performed. The CT detected destruction of two vertebral bodies, C7 and Th1, and a bony fragment - a remnant of C7 penetrated the trachea and probably repetitively punctured the inflated cuff (Figure 1).

**Figure 2:**
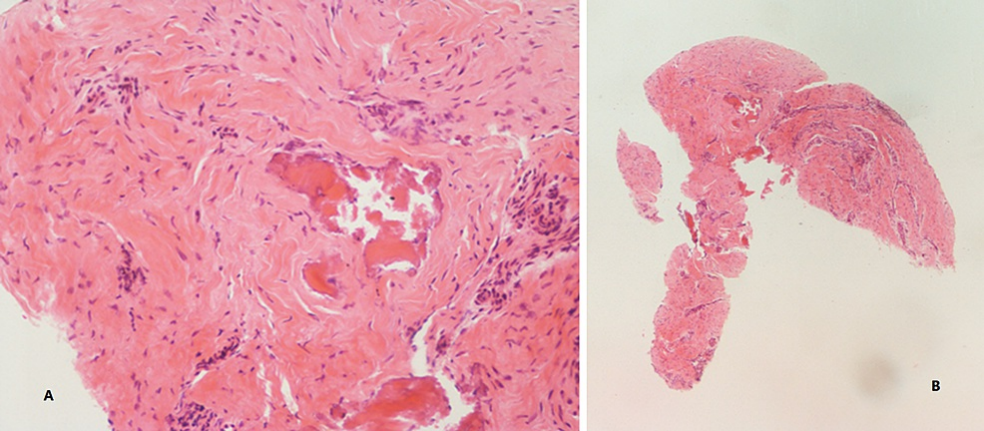
Histology of C7 vertebral body biopsy Necrotic bone spicules surrounded by a vascular-rich fibrous tissue, without evidence of inflammation, consistent with vertebral body avascular osteonecrosis (hematoxylin-eosin stain). (A) Magnification ×20; (B) magnification ×4.

Due to the patient’s general devastating condition, surgical intervention was rejected. The conservative management included the insertion of a new adjustable endotracheal tube (ET) with the aid of fiberoptic laryngoscopy. Besides, a biopsy of the C7 vertebral body was performed under CT guidance to rule out osteomyelitis. The biopsy revealed necrotic bone spicules surrounded by vascular-rich fibrous tissue, without evidence of inflammation (Figure 2). C7 vertebral body avascular osteonecrosis was diagnosed.

## Discussion

Effective tracheal occlusion plays an important role in preventing aspiration and increasing the efficiency of mechanical ventilation when the ET is inflated. However, high ET cuff pressure can damage the tracheal wall and lead to severe complications such as tracheomalacia and tracheal stenosis.

An ET cuff pressure of more than 30 cmH_2_O can impair capillary blood flow in the tracheal wall, and a cuff pressure of more than 50 cmH_2_O is considered high enough to completely block capillary blood flow [3]. However, even if cuff pressure is maintained below 30 cmH_2_O, it is still possible to alter tracheal venous and lymphatic pressures, which are 16 cmH_2_O and 4-6.5 cmH_2_O, respectively [4]. Low-pressure, high-volume ET cuffs are able to seal the trachea with low pressure and prevent these complications.

Given the repeated episodes of severe air leak with oxygenation drop due to cuff puncture, the attending physician decided to inflate the cuff with water, but the cuff pressure was not monitored. An overinflated tracheostomy tube cuff could be a cause of pathologic pressure on the trachea and anterior portion of the vertebral body.

In similar cases, conscious patients suffer significant discomfort from the tube, and sedatives and analgesics are titrated until the tube is tolerated. In the unconscious patient presented, this complication was not noted until a cause for recurrent puncture of the recurrent tracheostomy tube cuff was found.

The posterior two-thirds of vertebral bodies receive collateral blood from four arteries attained from two intervertebral levels, while the anterior third is defined as a “watershed zone,” thus having a higher risk of ischemic avascular necrosis [5-8]. Most probably, in our patient, prolonged mechanical pressure by water-hyperinflated tracheostomy cuff caused vertebral body ischemia and avascular osteonecrosis. 

## Conclusions

This case should be a lesson for those caring for patients with long-term cuff tracheostomy. The case highlights the importance of monitoring cuff pressure during long-term use of cuffed ETs to avoid hyperinflation and subsequent ischemic complications.
